# Gene Features Selection for Three-Class Disease Classification via Multiple Orthogonal Partial Least Square Discriminant Analysis and S-Plot Using Microarray Data

**DOI:** 10.1371/journal.pone.0084253

**Published:** 2013-12-30

**Authors:** Mingxing Yang, Xiumin Li, Zhibin Li, Zhimin Ou, Ming Liu, Suhuan Liu, Xuejun Li, Shuyu Yang

**Affiliations:** 1 Xiamen Diabetes Institute, the First Affiliated Hospital of Xiamen University, Xiamen, China; 2 Department of Endocrinology and Diabetes, the First Affiliated Hospital of Xiamen University, Xiamen, China; 3 Department of Electronic Science, School of Physics and Mechanical & Electrical Engineering, Xiamen University, Xiamen, China; King Abdullah University of Science and Technology, Saudi Arabia

## Abstract

**Motivation:**

DNA microarray analysis is characterized by obtaining a large number of gene variables from a small number of observations. Cluster analysis is widely used to analyze DNA microarray data to make classification and diagnosis of disease. Because there are so many irrelevant and insignificant genes in a dataset, a feature selection approach must be employed in data analysis. The performance of cluster analysis of this high-throughput data depends on whether the feature selection approach chooses the most relevant genes associated with disease classes.

**Results:**

Here we proposed a new method using multiple Orthogonal Partial Least Squares-Discriminant Analysis (mOPLS-DA) models and S-plots to select the most relevant genes to conduct three-class disease classification and prediction. We tested our method using Golub’s leukemia microarray data. For three classes with subtypes, we proposed hierarchical orthogonal partial least squares-discriminant analysis (OPLS-DA) models and S-plots to select features for two main classes and their subtypes. For three classes in parallel, we employed three OPLS-DA models and S-plots to choose marker genes for each class. The power of feature selection to classify and predict three-class disease was evaluated using cluster analysis. Further, the general performance of our method was tested using four public datasets and compared with those of four other feature selection methods. The results revealed that our method effectively selected the most relevant features for disease classification and prediction, and its performance was better than that of the other methods.

## Introduction

DNA microarray analysis is an important tool in medicine and life sciences, because it measures simultaneously the expression levels of thousands of genes. In the past few years, many multivariate data analysis methods have been developed and applied to extract the full potential from microarray experiments including cluster analysis [1], [2], support vector machine (SVM) [3], [4], self-organizing maps (SOMs) [5], [6], artificial neural networks (ANN) [7], [8], partial least squares (PLS) [9], and non-negative matrix factorization (NMF) [10], [11], [12], [13], [14]. Cluster analysis may be the most widely used method because, at least in part, it not only generates an intuitive tree to visualize clusters, and as an unsupervised technique, clusters samples and genes into different groups.

Microarray data typically consist of a relatively small sample size (usually several dozens) and a large number of genes (several thousands), most of which may be irrelevant, insignificant, or redundant for disease classification and prediction [15], [16]. Therefore, many gene-selection approaches for cluster analysis have been proposed such as signal to noise ratio (S2N) [5], ANN [7], Kruskal-Wallis nonparametric one-way ANOVA (KW) [17], ratio of genes between-categories to within-category sums of squares (BW) [18], nonparametric test [19], *t*-test [20], [21], genetic algorithm (GA), and k-nearest neighbor (GA/KNN) [22]. Many approaches were aimed to deal with two-class gene selection problems, and only a few studies involved multiclass gene selection (three classes or more) and classification for cluster analysis.

In this study, we describe the development of a novel multiple orthogonal partial least squares discriminant analysis (mOPLS-DA) model and S-plots to conduct three-class gene selection. Partial least squares (PLS), a well-known dimension-reduction method, is a simple and efficient algorithm that conducts two-class and multiclass classification [9], [23] and predicts clinical outcome [24] and survival times [25] using gene expression microarray data. When the response is dummy variable (0/1), the PLS model is called partial least squares discriminant analysis (PLS-DA) [26]. OPLS-DA is a new version of PLS-DA with built-in orthogonal signal correction (OSC) [27]. PLS-DA and OPLS-DA are widely used for analyzing data of gene expression [9], [23], [24], [28], proteomics [29], [30], [31], and metabolite profiling [32], [33], [34], [35], [36]. A new feature of OPLS-DA is S-plot [37], which is widely used in MS-based metabolomics studies [38], [39]. OPLS-DA and S-plots are typically used to identify significant biomarkers to distinguish between two groups. Here we employed two hierarchical OPLS-DA models with hierarchical S-plots as well as three parallel OPLS-DA models with multiple S-plots to select informative genes for three classes with subtypes and three classes in parallel, respectively. We demonstrate our approach using Golub’s leukemia dataset [5] and evaluated its general performance using four other publicly available datasets. The performance of feature selection for disease classification and prediction was visualized by the tree plot of cluster analysis.

## Materials and Methods

### Data Resource

We used Golub’s leukemia microarray expression dataset [5] because it is widely used to evaluate the performance of new algorithms to identify gene markers or to classify different kinds of cancers. Golub’s dataset contains 72 tissue samples, 38 observations in a training set comprising 19 ALL-B, 8 ALL-T, and 11 AML subjects as well as 34 samples in an independent test set, including 19 ALL-B, 1 ALL-T, and 14 AML subjects. Affymetrix high-density oligonucleotide microarray, containing 7129 probes for 6817 human genes, was detected. There were wide differences between training and test set [5]. For example, samples from different reference laboratories were prepared using different experimental protocols and subjects were adult patients with AML in the training set, and adults and children with AML in the test set.

We chose this dataset, because it is publicly available and has been analyzed by many others. Further, this dataset comprises the two main classes of ALL and AML, and ALL can be classified into ALL-B and ALL-T subtypes. We first treated this dataset as three classes with subtypes and then as three parallel classes.

### Data Preprocessing and Scaling

The microarray dataset was preprocessed following Golub’s preprocessing steps [40] as follows: thresholding, setting the minimum (min) and maximum (max) expression values to 100 and 16000, respectively; filtering, discarding genes with max/min ≤5 or (max-min) ≤500; and log_10_-transformation. Each preprocessed variable was scaled by centering for principal component analysis (PCA) [41], OPLS-DA, and cluster analysis. Centering scaling was selected, because the deviation of the preprocessed variables was in a limited range after log transformation.

### PCA

PCA is a multivariate projection method [42]. The objective of PCA is to project each observation onto a subspace to extract maximum variation from the data matrix. It reduces thousands of dimensionalities of the data matrix to a small number, for example, 3 or 5. The information of observations and variables can be visualized in two- or three-dimensional scatter plots called the score plot and loading plot, respectively. The first principal component models the largest variation and the second component captures the second-largest variation from the dataset, and so on. The score plot of PCA provides overviews, trends, groupings, and outliers of the observations [43]. The PCA model can be expressed as follows:
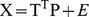



Where T is the score vector, P is the loading vector, and *E* describes the residual matrix that was not fitted in the PCA model.

### OPLS-DA

OPLS-DA [26] is a new version of PLS. PLS regression [44], [45] is a recently developed multivariate algorithm of multiple linear regression which relates numerous response variables (Y) to X blocks of matrices by a linear multivariate model. It is more practical and robust than traditional least squares regression methods, because it effectively handles colinearity of variables, noise in both blocks of variables, missing data, and the question that the number of variables is larger than that of samples. Orthogonal partial least squares (OPLS), a minor modification of the PLS algorithm, which was introduced by Trygg [27], possesses built-in orthogonal signal correction (OSC) [46] that filters out some variance in the X-matrix unrelated to Y. The OPLS model separates systematic variation in the X-block into two parts, called predictive and orthogonal, respectively. The first fits the covariance between X and Y, and the second contains systematic variation in X that is unrelated to Y. When Y is constructed using dummy variable (0/1), PLS and OPLS are called PLS-DA and OPLS-DA, respectively. Compared with PLS-DA, OPLS-DA is more effective in focusing the correlated information onto the first predictive component instead of scattering them on the subsequent components. Hence, the main benefit of OPLS-DA is that its results are clearer and easier to interpret. The OPLS algorithm has been described in detail [27], [47]. In this study, PCA and OPLS-DA analyses of microarray data were implemented using SIMCA-P+12.0 software (Umetrics AB, Sweden).

### Gene Feature Selection using S-plot

The purpose of S-plot is to select putative interesting variables from the X-matrix based on OPLS-DA model. One advantage of the S-plot derived from the predictive component of OPLS-DA is that it combines the contributions and reliability of variables in a scatter plot. That is, the x-axis of the S-plot describes the fitted covariance vector, cov(t, X), and the y-axis represents the correlation coefficient vector, corr(t, X). The two vectors of the S-plot are calculated as follows [37]:
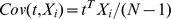


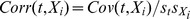



Where *t* is the score vector in the OPLS-DA model predictive component, *s_t_* is the standard deviation of the OPLS predictive score vector, and *s_X_* is the estimated standard deviation vector for each variable. This combination is particularly important, because it facilitates determination of variables with the highest correlation coefficient and the largest contribution to the model separation between two classes.

The number of variables selected as potential markers is application-dependent, but the rule of thumb is recommended. In the present study, following Golub’s paper [5], the top-ranked 50 genes were selected as putative gene markers according to their correlation (p(corr)) and contribution coefficients (p).

### Evaluation of the OPLS-DA Model

The goodness of fit and prediction of the OPLS-DA model were evaluated using model parameters R^2^Y and Q^2^Y, respectively, according to the previous literature [42], [48]. Q^2^Y was calculated as follows:
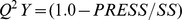



Where PRESS is the prediction error of the sum of squares and SS is the sum squares of the response variables. The statistical significance of the OPLS-DA model was evaluated using the cross validation-analysis of variance (CV-ANOVA) technique [49].

### Evaluation of the Performance of Selected Genes using Cluster Analysis

Cluster analysis was used to test the rationality of the selected gene markers. For cluster analysis, the Euclidean or Pearson distance was chosen to compute the distance of dissimilarity using software PermutMatrixEN [50] (Version 1.9.3, http://www.lirmm.fr/~caraux/PermutMatrix/).

### Evaluation of the General Performance of Feature Selection of mOPLS-DA for Three Classes in Parallel using Four Public Datasets

Four publicly available datasets, including 11_tumour [51], leukemia_2 [52], 14_tumour [53], and SRBCT [7] were used to evaluate the general performance of our method, because these datasets all comprise training and test sets that were defined in the their original publication, and their sample sizes are relatively large. These datasets were used to evaluate the performance of multicategory support vector machines (MC-SVMs) for cancer diagnosis [17]. Three classes with the largest sample size in these datasets were selected for further analysis. All datasets were preprocessed according to the descriptions in the primary studies [7], [51]–[53]. We also used four feature selection methods such as BW [40], one-versus-rest S2N (OVR-S2N) [5], KW [17], and OVR-*t*-test as references to test the general performance of our method. OVRSVM [17] was used as classifier as well. The GEMS system [17] and spider software (http://people.kyb.tuebingen.mpg.de/spider/) in the MATLAB environment were employed to filter genes and fit classification model of OVRSVM.

We first used these methods to extract features from the training set and utilized them to fit the classification models of SVM and cluster analysis. Observations in the training and independent test sets were classified and predicted using the classification models. The number of misclassified observations was counted.

## Results

### Overview the New ALL-AML Training and Test Sets

**Figure 1:**
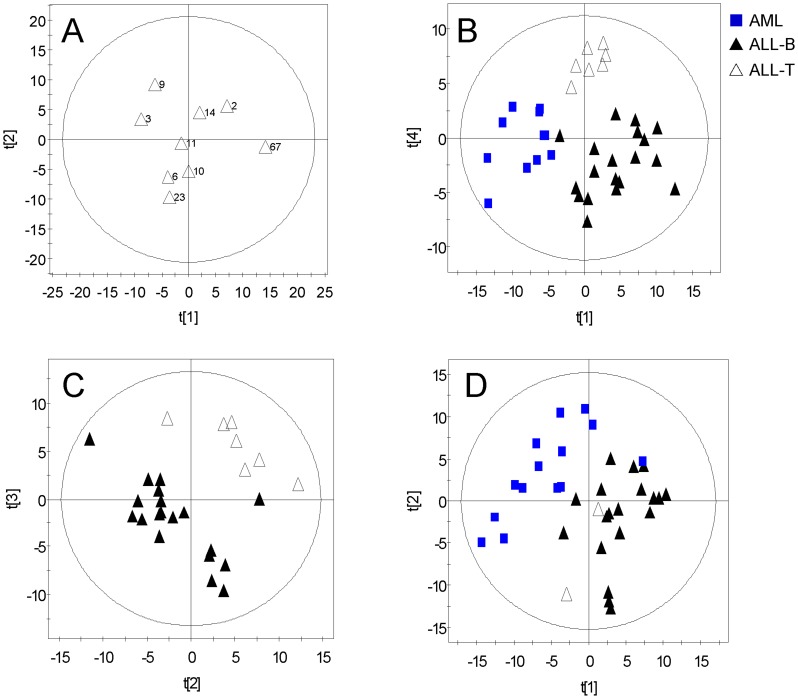
PCA score plots of the new training and test sets. A, PCA score plot of all ALL-T samples. From this plot, we selected two informative samples (# 9 and 10) as new test samples for the ALL-T class. B, Score plot of the first and fourth principle component from PCA of the new training set. C, PCA score plot of second and third components from ALL-B and ALL-T. D, PCA score plot of the new test set.

After data preprocessing, 3571 genes remained. Because Golub’s test set included only one ALL-T (T-cell ALL) sample (**#**67), it was difficult to assign this sample to one class. Therefore, we selected two representative samples (**#**9, 10) by performing PCA on all ALL-T samples from the training and test sets (Figure 1A) and included them with the rest of the original test set to form a new independent test set according to the design of experimental (DOE) [54]. PCA was then conducted using the new training and test sets, respectively, to generate overviews of the observations. The score plots of the training set showed a clear separation among three classes of ALL-B (B-cell ALL), ALL-T, and AML on the first and fourth principal components (Figure 1B), and less separation for the test set using the first two components (Figure 1D). No strong outliers were found in training and test set. A distinct difference in gene profiles between ALL-B and ALL-T was also observed in the PCA score plot of ALL-B and ALL-T (Figure 1C).

### OPLS-DA Model of Three Classes with Subtypes

Because ALL-B and ALL-T are subtypes of acute lymphoblastic leukemia (ALL), we first regarded Golub’s leukemia data as a three-class disease with subtypes. Therefore, we fitted two OPLS-DA models using the training set (Table 1). The first model was constructed using AML against all ALL samples, including ALL-B and ALL-T, to select characteristic genes that distinguished AML from ALL. The second OPLS-DA model was fitted with ALL-B and ALL-T samples of the training set to identify the most significant genes for each subtype. The first OPLS-DA model extracted one predictive and two orthogonal components from data matrix. The percentage of extracted variance related to class information was only 30.3% but explained 97.8% of the variance of class variables (0/1) (R^2^Y = 0.978). This OPLS-DA model had good predictive power as indicated by the goodness of the prediction parameter (Q^2^Y = 0.798) computed using cross-validation (Table 1). The difference between R^2^Y and Q^2^Y was only 0.18. The CV-ANOVA test showed that the model was significant (P = 3.28488e-009). Score plots of the first OPLS-DA model revealed a clear separation was achieved along the first predictive component (data not shown).

**Table 1:** OPLS-DA models of three classes with subtypes and three classes in parallel using the training set.

		OPLS-DA Models	R^2^X	R^2^Y	Q^2^Y	Increased	Decreased
Three classes with subtype	Model 1	AML vs ALL-T and ALL-B	0.303	0.978	0.798	20 in AML	20 in AML
	Model 2	ALL-B vs ALL-T	0.348	0.99	0.806	5 in ALL-B	5 in ALL-B
Three classes in parallel	Model 1	AML vs ALL-T and ALL-B	0.303	0.978	0.798	11 in AML	6 in AML
	Model 2	ALL-B vs AML and ALL-T	0.275	0.984	0.823	12 in ALL-B	5 in ALL-B
	Model 3	ALL-T vs AML and ALL-B	0.271	0.983	0.804	12 in ALL-T	5 in ALL-T

Keys: R^2^X, the cumulative fraction of Sum of Squares (SS) of X explained by components; R^2^Y, the cumulative Sum of Squares of all the y-variables explained by the extracted components; Q^2^Y, The fraction of the total variation of Y (PLS and OPLS) that can be predicted by the extracted components.

The parameters of the goodness of fit and prediction of the second OPLS-DA model showed that this model was good (R^2^X = 0.348, R^2^Y = 0.99, Q^2^Y = 0.806) (Table 1). The P-value of the CV-ANOVA of the second OPLS-DA model was also significant (P = 7.0255e-006). The score plot of the second OPLS-DA model exhibited a complete separation between ALL-B and ALL-T on the first predictive component (data not shown).

### Gene Selection for Three Classes with Subtypes

**Figure 2:**
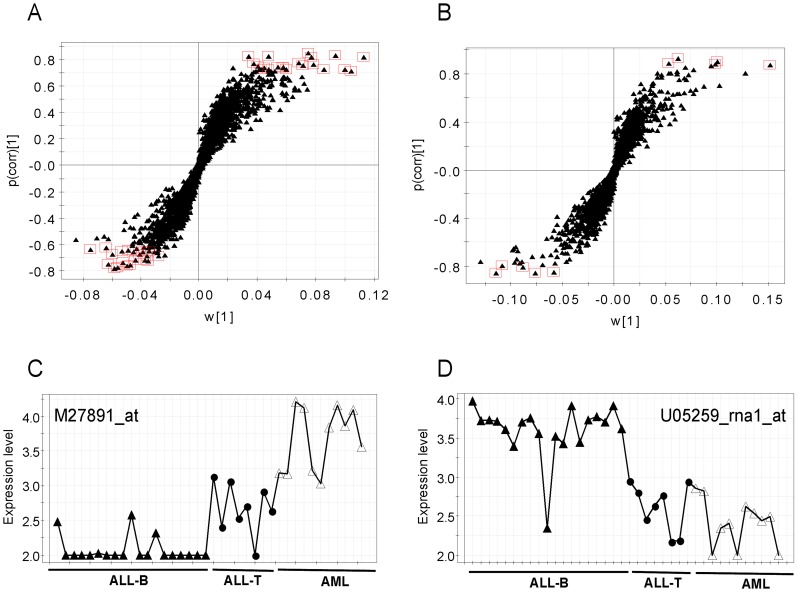
S-plots for gene selection of three classes with subtypes. A, S-plot from the OPLS-DA model of AML and ALL. Twenty genes with increased and decreased levels, respectively, in AML were selected from this S-plot. B, S-plot of OPLS-DA of ALL-B and ALL-T. From this S-plot, five genes with elevated and reduced levels in ALL-B, respectively, were selected. C, A typical gene (M27891_at) with a profile of increased expression levels in AML and decreased levels in ALL-T and ALL-B. D, The levels of gene U05259_rna1_at upregulated in ALL-B and downregulated in ALL-T. Key: p(corr) [1] and w [1] are the correlation coefficient and contribution coefficient vectors of the predictive component of the OPLS-DA model.

Model evaluation parameters suggested that the two OPLS-DA models were reliable. Two S-plots from these OPLS-DA models were employed for feature selection, and the top-ranked 50 genes were selected from 3571 genes as the most informative. We selected 40 genes with the largest correlation and covariance related to model class information using the first OPLS-DA model in which the expression levels of 20 genes increased and 20 others decreased in the AML group (Figure 2A). Thus, the expression levels of 20 genes decreased and the other 20 genes increased in the ALL group (ALL-B and ALL-T). A typical gene with elevated and decreased levels in AML and ALL, respectively, is shown in Figure 1C. Ten genes were chosen from the S-plot of the second OPLS-DA model. Here, the expression levels of five genes were elevated and the other 5 decreased in ALL-T (Figure 2B). Figure 2D shows the plot of a selected gene with increased and decreased levels in ALL-B and ALL-T, respectively.

### Validation of the Top 50 Genes for Three Classes with Subtypes

**Figure 3:**
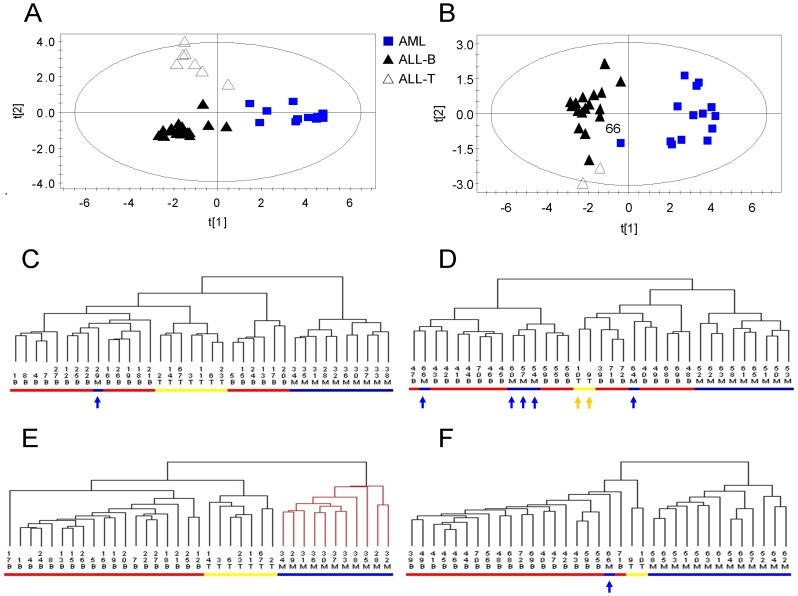
PCA score plot and cluster analysis tree plot of training and test sets. A, PCA score plot of the training set using the top 50 genes. B, PCA score plot of the test set of the top 50 genes. C, Cluster analysis tree plot of the training set of the initial 3751 genes. #29 (in blue mark) was misclassified. D, Cluster analysis tree plot of the test set with 3751 genes. #54, 57, 60, 66 (in blue mark) and #9, 10 (in yellow mark) were misclassified. Cluster analysis trees of C and D were computed using the Euclidean distance and Ward’s linkage. E, Cluster analysis tree plot of the training set of the top 50 genes. F, Cluster analysis the tree plot of the test set of the reduced top 50 genes. Only #66 (in blue mark) was misclassified. Cluster analysis of E and F were carried out using the Euclidean distance and average linkage. Keys for C, D, E, F: B, ALL-B (red); T, ALL-T (yellow); M, AML, (blue).

To evaluate the classification performance of the top-ranked 50 genes, we performed PCA on reduced training and test sets of 50 genes selected above. The PCA score plot of the reduced training set showed that AML, ALL-B, and ALL-T were completely separated and localized to three regions (Figure 3A). PCA of reduced test set separated the three groups except for #66 (Figure 3B). Cluster analysis was used to visualize the classification power of these 50 genes. The cluster tree of the reduced training set showed that all samples were correctly classified (Figure 3E). Notably, the subtypes of ALL-B and ALL-T were also accurately classified (Figure 3E). Although we selected the best results of clustering for the training set with 3571 genes, one AML sample was misclassified into the ALL-B group (#29) and ALL was misclassified into three subclasses (Figure 3C).

The results of cluster analysis showed that the classification performance of the test set comprised of top 50 genes was excellent, because only one sample was misclassified (#66) (Figure 3F). This sample was incorrectly assigned to the ALL group by Golub [5] and other researchers [9], [55], [56]. Moreover, two ALL-T samples (#9, 10) were grouped together in one class and parallel with the ALL-B group (Figure 3F). With the 3571 gene dataset, AML and ALL were not clearly distinguished, and two ALL-T samples were incorrectly predicted as ALL-B together with the AML samples (Figure 3D).

### Feature Selection for Three Parallel Classes

**Figure 4:**
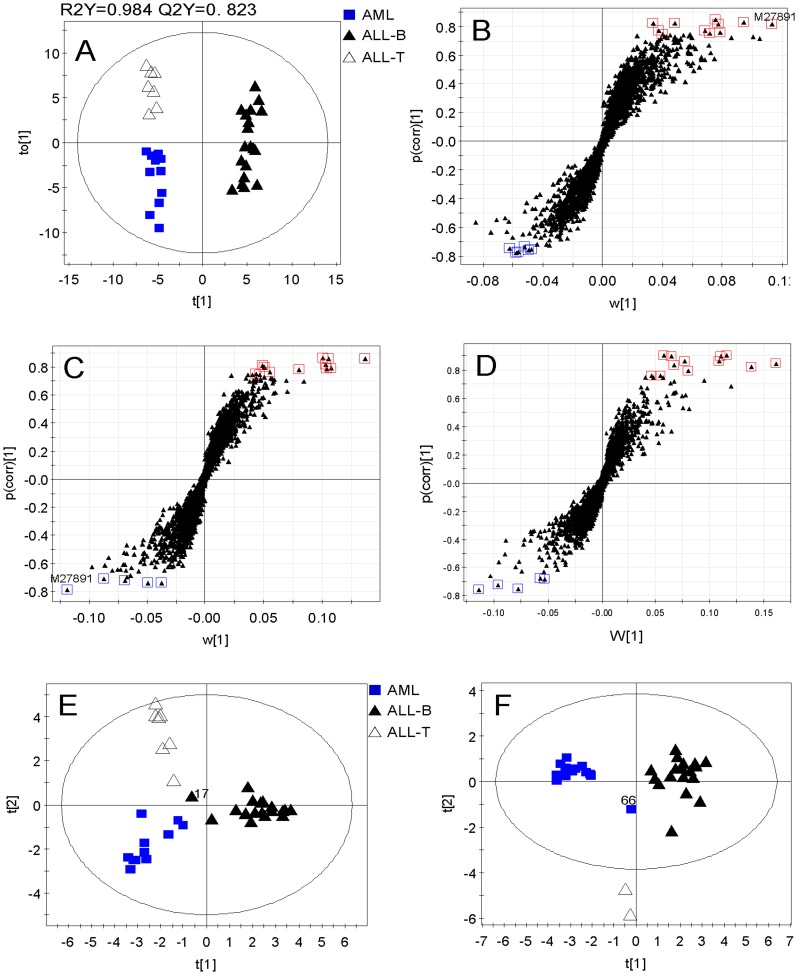
A, OPLS-DA score plot of ALL-B vs. AML and ALL-T. B, OPLS-DA S-plot of AML vs. ALL-B and ALL-T, eleven and six up- and down-regulated genes, respectively, in AML were selected from this S-plot. C, OPLS-DA S-plot of ALL-B vs. AML and ALL-T. Twelve and five up- and downregulated genes, respectively, in ALL-B were selected. D, OPLS-DA S-plot of ALL-T vs. ALL-B and AML. Twelve and five up- and downregulated genes, respectively, in ALL-T were selected. E, PCA score plot of the training set with top 50 genes. F, PCA score plot of the test set with the selected top 50 genes.

We next considered AML, ALL-B, and ALL-T as three parallel classes without subtypes to select characteristic genes for classifying disease. Therefore, we selected features for each class through the corresponding OPLS-DA models and S-plots. Three OPLS-DA models were fitted using training set of AML vs. ALL-B and ALL-T, ALL-B vs. AML and ALL-T, and ALL-T vs. AML and ALL-B (Table 1). The parameters of model evaluation showed that these three models were very good in the goodness of fit and prediction (Table 1). Score plots of each OPLS-DA model demonstrated that each group was clearly separated from the others on the first predictive component. Figure 4A is the score plot from OPLS-DA model of ALL-B vs. AML and ALL-T which shows that ALL-B is distinct from AML and ALL-T, and more interestingly, AML is separated from ALL-T on the first orthogonal component.

Seventeen top genes were selected from each OPLS-DA model using the S-plot (Figure 4B, C, D). The number of genes selected from each model and the model parameters are shown in Table 1. Note that feature selection depended mainly on the correlation between gene variables and the predictive scores p(corr) and that the genes with a larger contribution were preferred when there was no significant difference in the correlation between two genes. Among them, gene M27891 was chosen twice. Hence, only the top-ranked 50 genes were selected and analyzed further. We next performed PCA on the training and test sets with the new top-ranked 50 genes. The PCA score plot of the training set showed that three classes clustered in three distinct regions except one sample (#17), which was in the joint region (Figure 4E), and the score plot of the test set showed that three classes were clearly located in three regions of the score plot with an unknown-class sample (#66) (Figure 4F).

### The Classification Power of the Top 50 Genes for Three Parallel Classes

**Figure 5:**
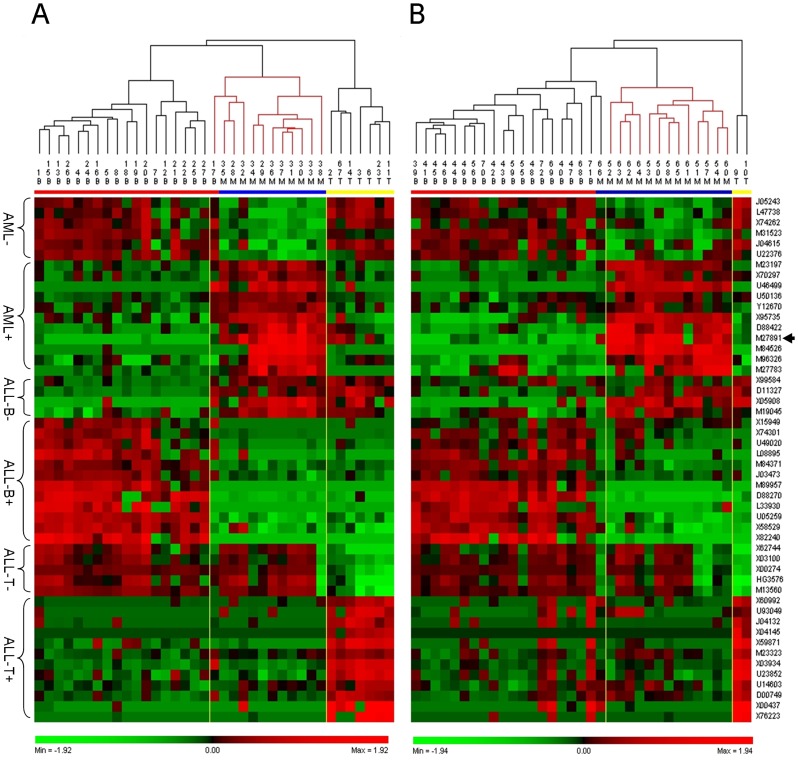
Cluster analysis tree plot of the reduced training and test sets of the top 50 genes. A, Training set. B, Test set. Cluster analysis was computed using the Euclidean distance and complete linkage. Keys: B, ALL-B (red); T, ALL-T (yellow); M, AML (blue). AML- and AML+ indicate gene up- and downregulated expression levels in AML samples, respectively; ALL-B- and ALL-T+ represent down- and upregulated expression levels in ALL-B, respectively; ALL-T− and ALL-T+ indicate ALL-T samples with down- and upregulated expression levels, respectively.

To examine the rationality of the top 50 genes, we next performed cluster analysis on the training and test sets, respectively, using the Euclidean distance and complete linkage. The cluster tree for the training set of the new top 50 genes showed that all samples were assigned accurately into ALL-T, ALL-B, and AML, except for one sample (#17), which belongs to the ALL-B group but was incorrectly classified as AML (Figure 5A). We found that when we separated all training samples into two classes, these two clusters were ALL-T vs. ALL-B and AML other than AML vs. ALL-B and ALL-T. Similarly, all test set samples clustered into three distinct classes in a profile similar to that of the training set with one misclassified sample (#66). There were only two samples in the ALL-T group of the test set (#9, 10). However, these two samples still clustered into one class in parallel with ALL-B and AML (Figure 5B). The gene features identified by each model were displayed in a color-coded data matrix in Figure 5A and B, and there was no difference in the gene expression profile between training and test sets.

### Comparison of the Classification Performance with t-test

**Figure 6:**
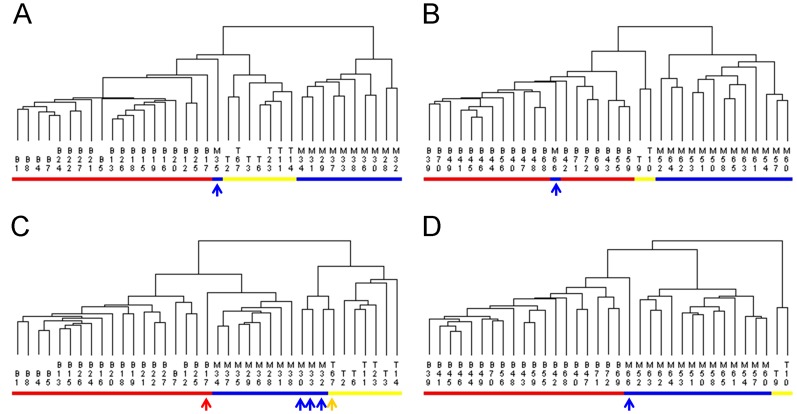
Cluster analysis plots of OVR-*t*-test results with the top 50 genes based on P value for three classes with subtypes (A and B) and three classes in parallel (C and D) for training set (A and C) and the independent test set (B and D). For three classes with subtypes, two samples (#35, 66) were misclassified (blue arrow, panels A and B) using the top 50 genes selected by the OVR-*t*-test. For three classes in parallel, six observations (#17, 30, 32, 33, 67, and 66) were misclassified (red, blue, and yellow arrows in panels C and D). Key: B, T, M denote ALL-B (red), ALL-T (yellow), and AML (blue), respectively.

To assess the performance of our approach, we compared the classification power of mOPLS-DA with that of *t*-test using Golub’s dataset. The OVR-*t*-test was employed to select significant features for each class. Selection methods were described in Table 2. For three classes with subtypes, one sample each from the training and test sets was misclassified (Figure 6A and B). Unfortunately, there were six misclassified observations (five from the training set and one from test set) for three classes in parallel (Figure 6C and D).

**Table 2:** Feature selection of the top genes for the OVR-*t*-test.

	OVR-*t*-test	Top P-value genes	With increased level
Three classes with subtype	AML vs ALL-T and ALL-B	40	32 in AML
	ALL-B vs ALL-T	10	9 in ALL-B
Three class	AML vs ALL-T and ALL-B	17 in AML	6 in AML
	ALL-B vs AML and ALL-T	17 in ALL-B	13 in ALL-B
	ALL-T vs AML and ALL-B	17 in ALL-T	2 in ALL-T

### General Performance of Feature Selection of Our Proposed Methods

Table 3 shows the results for the selection performance of the five gene-filter methods. Using SVM as the classifier, the BW, S2N-OVR, KW, OVR-*t*-test methods all performed well and our method outperformed them with no misclassification. When cluster analysis was used as the classifier, the performance of BW, S2N-OVR, KW, and the OVR-*t*-test became weaker, particularly for the SRBCT dataset, whereas the performance of mOPLS-DA was the same as that of SVM (Table 3 and Supplementary Figure S1, S2, S3, S4). Further, we found that the performance of the OVR-*t*-test was better than that of the others, except mOPLS-DA.

**Table 3:** General performance of mOPLS-DA using four datasets.

Classifier/featureselection methods		11_tumour [51](BR/OV/PR)		Leukemia_2 [52](ALL/AML/MLL)		14_tumour [53] (Leukemia/Lymphoma/CNS)		SRBCT [59](RMS/EMS/NB)	
		Train set(misclassified/total)	Test set(misclassified/total)	Train set(misclassified/total)	Test set(misclassified/total)	Train set(misclassified/total)	Test set(misclassified/total)	Train set(misclassified/total)	Test set(misclassified/total)
OVR^b^SVM	BW	0/31^a^	2/48	0/57	0/10	0/56	3/16	0/55	0/17
	OVR-S2N	0/31^a^	0/48	0/57	0/10	0/56	3/16	0/55	0/17
	KW^c^	0/31	1/48	0/57	0/10	0/56	1/16	0/55	0/17
	OVR-t-test	0/31	0/48	0/57	1/10	0/56	3/16	0/55	1/17
	mOPLS-DA	0/31	0/48	0/57	0/10	0/56	0/16	0/55	0/17
CA^d^	BW	2/31	2/48	3/57	0/10	5/56	2/16	26/55	6/17
	OVR-S2N	1/31	4/48	1/57	0/10	10/56	2/16	23/55	7/17
	KW^c^	3/31	3/48	5/57	1/10	2/56	1/16	23/55	7/17
	OVR-t-test	2/31	2/48	3/57	0/10	0/56	1/16	1/55	4/17
	mOPLS-DA	0/31	0/48	1/57	0/10	0/56	0/16	0/55	0/17

Features were selected from the training set to fit OVRSVM models using the individual feature selection method. Observations of the training and independent test sets were classified and predicted by the corresponding fitted model, and the number of misclassified observation was counted.

Key: ^a^the number of features was optimized from 100 to 300 with the step of 10 and set to 160;

^b^ OVR, *one-versus-rest*;

^c^ KW, Kruskal–Wallis non-parametric one-way ANOVA;

^d^ cluster analysis.

## Discussion

In the present study, we developed a novel three-class gene selection method for disease classification and prediction using mOPLS-DA models and S-plots to identify informative genes from microarray data. The rationale for using mOPLS-DA and S-plots to select gene features to classify and predict three-class disease is that differentially expressed genes for each class should be selected using the corresponding OPLS-DA model and S-plots. Unlike other studies that validate the performance of feature selection by leave-one-out cross-validation (LOOCV) using only one dataset, we evaluated our method using internal cross-validation (model evaluation parameters) and external validation with an independent data set. Comparative results from Golub’s leukemia data and four public datasets showed that mOPLS-DA and S-plots worked better for selecting the most relevant genes for three-class classification and prediction than the other four feature selection methods. As suggested in Table 3, our method achieved perfect classification and prediction independently of the classifier.

Golub’s leukemia data was used to evaluate the performances of Bayesian model averaging (BMA) [56] and multicategory support vector machine (MSVM) [55] for the three-class problem in parallel. The predictive power of our method tested using cluster analysis was similar to that of BMA and MSVM with only one misclassified observation (#66). Unlike these studies [55], [56], in the present study we did not optimize the number of genes used for classification and prediction. Therefore, we conclude that the performance of our method is at least similar to those of these techniques for classifying and predicting three-class disease. Moreover, the *t*-test, which is a standard and widely used method for feature selection, outperformed the other gene-selection approaches, including BW, KW, and OVR-S2N. This finding is consistent with Haury’s report [57].

When ALL-B, ALL-T, and AML were considered as three parallel classes, the clustering dendrogram revealed that the gene expression pattern of ALL-B was much closer to that of AML compared with ALL-T (Figure 5A, B), which is consistent with previous results [55]. To maintain the shape of class subtypes, we proposed a hierarchical OPLS-DA model and used S-plots to select the most relevant genes for three classes with subtypes. The clustering trees shown in Figure 3E, F indicated that this method was advantageous for choosing the most closely related genes that maintain the original subtypes of the disease classes.

OPLS-DA is a scaling-dependent chemometrics approach. Three scalings are available for OPLS-DA such as centering, unit variance (UV), and pareto. Centering scaling increases the influence of variables with larger amplitudes on the model. UV (autoscaling) is the most commonly used alternative scaling technique that centered each variable and then divided by its standard deviation. The disadvantage of UV is that it often inflates the importance of noise, which may mask the variables of interest. Pareto scaling is a compromise between center and UV scaling. For generating S-plots from OPLS-DA, centering and pareto are available. Because log_10_-transformation of data in preprocessing made the ranges of gene expression levels in an acceptable limit, the centering scaling was employed in PCA, OPLS-DA, and cluster analysis. Further, for pareto scaling, the classification accuracy of reduced training and test datasets was lower than that of centering scaling (data not shown).

The PLS and OPLS models are fitted using a strategy that extracts components from matrix X, which is different from traditional regression modeling that depends on covariance decomposition. The robustness of PLS and OPLS models are not affected by multi-colinearity of variables and not constrained by larger number of variables than that of the observations [58]. It is possible for OPLS-DA and S-plot to identify the variables with the greatest predictive power, because it is not necessary to filter out highly correlated variables before model construction. Another advantage of the OPLS-DA model is that this model rarely overfitted [27], because only one predictive component is used to fit the regression model for OPLS-DA.

## Supporting Information

Figure S1 **Heatmaps of cluster analysis of top 51 genes selected by mOPLS-DA models using training set (A) and test set (B) of SRBCT dataset.** Three classes including RMS, EMS and NB were used in current work. No observation was misclassified by cluster analysis of top51 genes selected by our proposed method. In original work, test sample 1, 8, 14, 16, 23 and 25 were diagnosed as NB; test 2, 6, 12, 19, 20 and 21 was diagnosed as EWS by histological examination; test 4, 10, 17, 22 and 24 belonged to RMS.
(TIF)

Figure S2 **Plots of cluster analysis of reduced training set (A) and test set (B) consisting of top 51genes selected from 11_tumour dataset by mOPLS-DA models.** No observations were misclassified in training set and wrongly predicted in test set.
(TIF)

Figure S3 **Heatmaps from cluster analysis of reduced training set (A) and test set (B) of top 51 genes chose from dataset of Leukemia_2 by mOPLS-DA models.** In training set, only one observation (MLL 17) was misclassified; all observations in independent test set were predicted correctly.
(TIF)

Figure S4 **Cluster analysis plot from reduced training set (A) and test set (B) contained top 51 genes selected by mOPLS-DA models using 14_tumour dataset.** From these two figures, we can see that all training samples were classified correctly and all test observations were predicted into each class without a mistake.
(TIF)
