# Supplementary figures and images for: Gene Features Selection for Three-Class Disease Classification via Multiple Orthogonal Partial Least Square Discriminant Analysis and S-Plot Using Microarray Data
Source: PLoS One. 2013 Dec 30;8(12):e84253. doi: 10.1371/journal.pone.0084253 (PMC3875537; doi:10.1371/journal.pone.0084253)

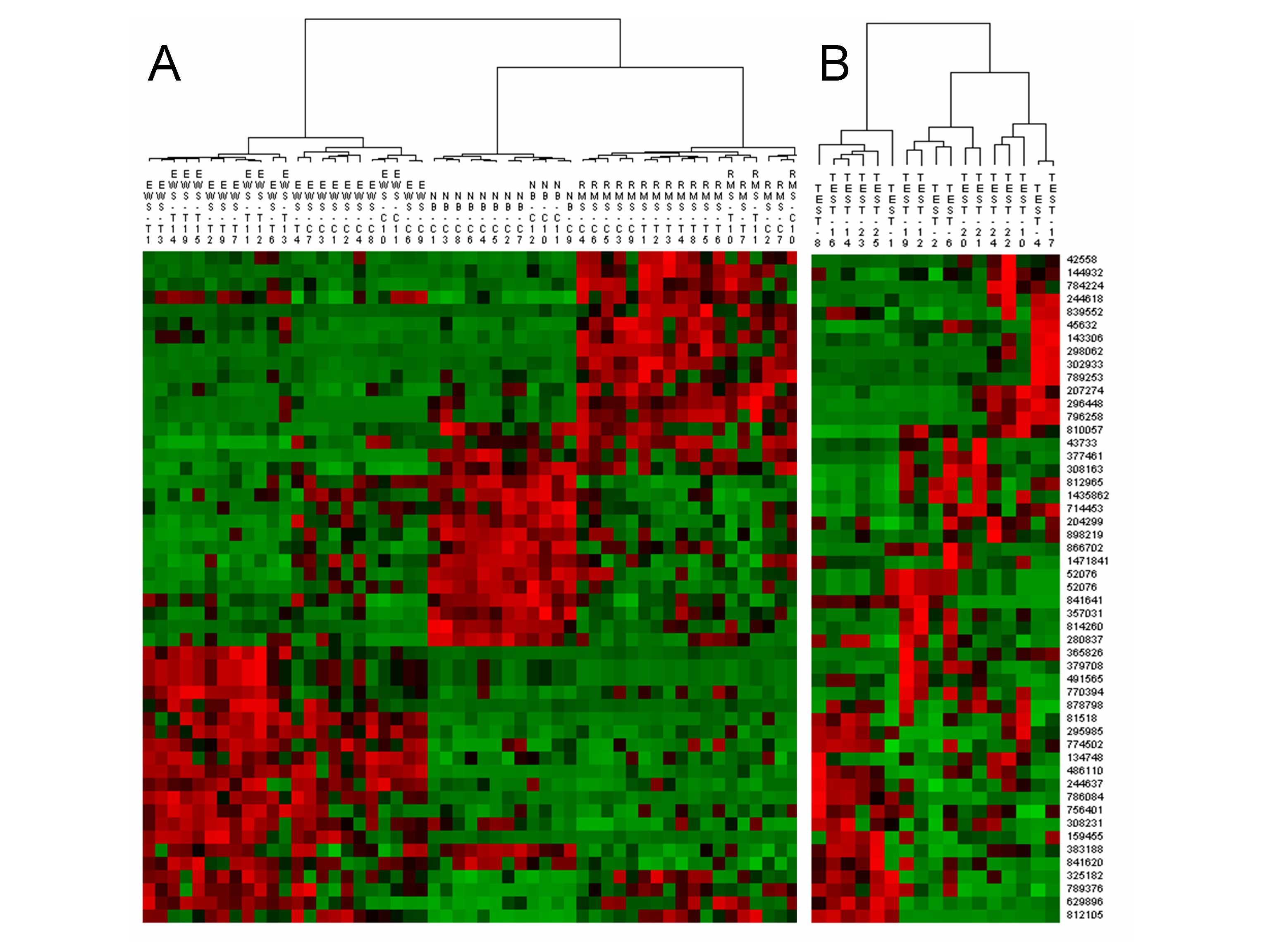

Supplement: Figure S1 — Heatmaps of cluster analysis of top 51 genes selected by mOPLS-DA models using training set (A) and test set (B) of SRBCT dataset. Three classes including RMS, EMS and NB were used in current work. No observation was misclassified by cluster analysis of top51 genes selected by our proposed method. In original work, test sample 1, 8, 14, 16, 23 and 25 were diagnosed as NB; test 2, 6, 12, 19, 20 and 21 was diagnosed as EWS by histological examination; test 4, 10, 17, 22 and 24 belonged to RMS. (TIF) [file pone.0084253.s001.tif]

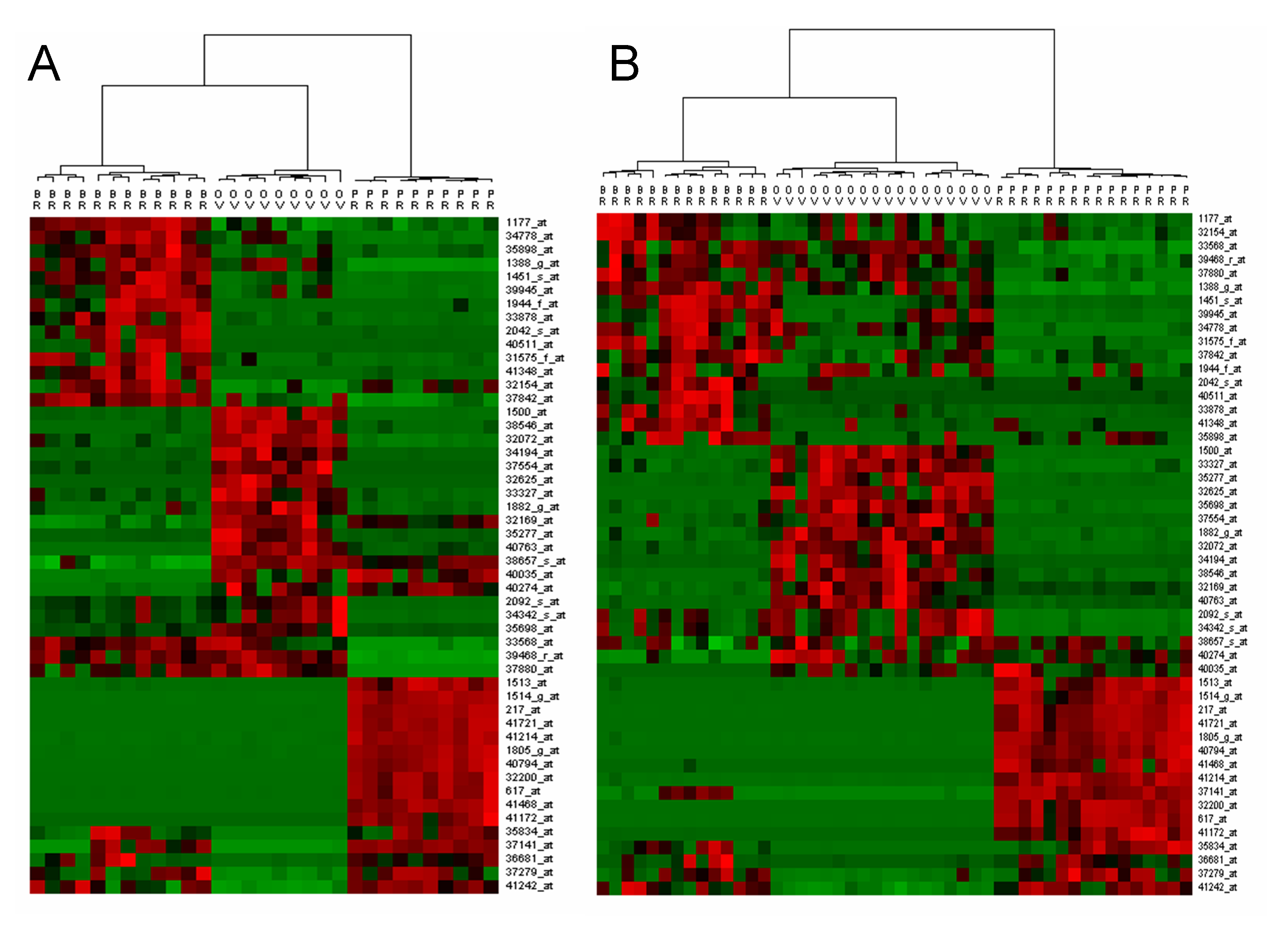

Supplement: Figure S2 — Plots of cluster analysis of reduced training set (A) and test set (B) consisting of top 51genes selected from 11_tumour dataset by mOPLS-DA models. No observations were misclassified in training set and wrongly predicted in test set. (TIF) [file pone.0084253.s002.tif]

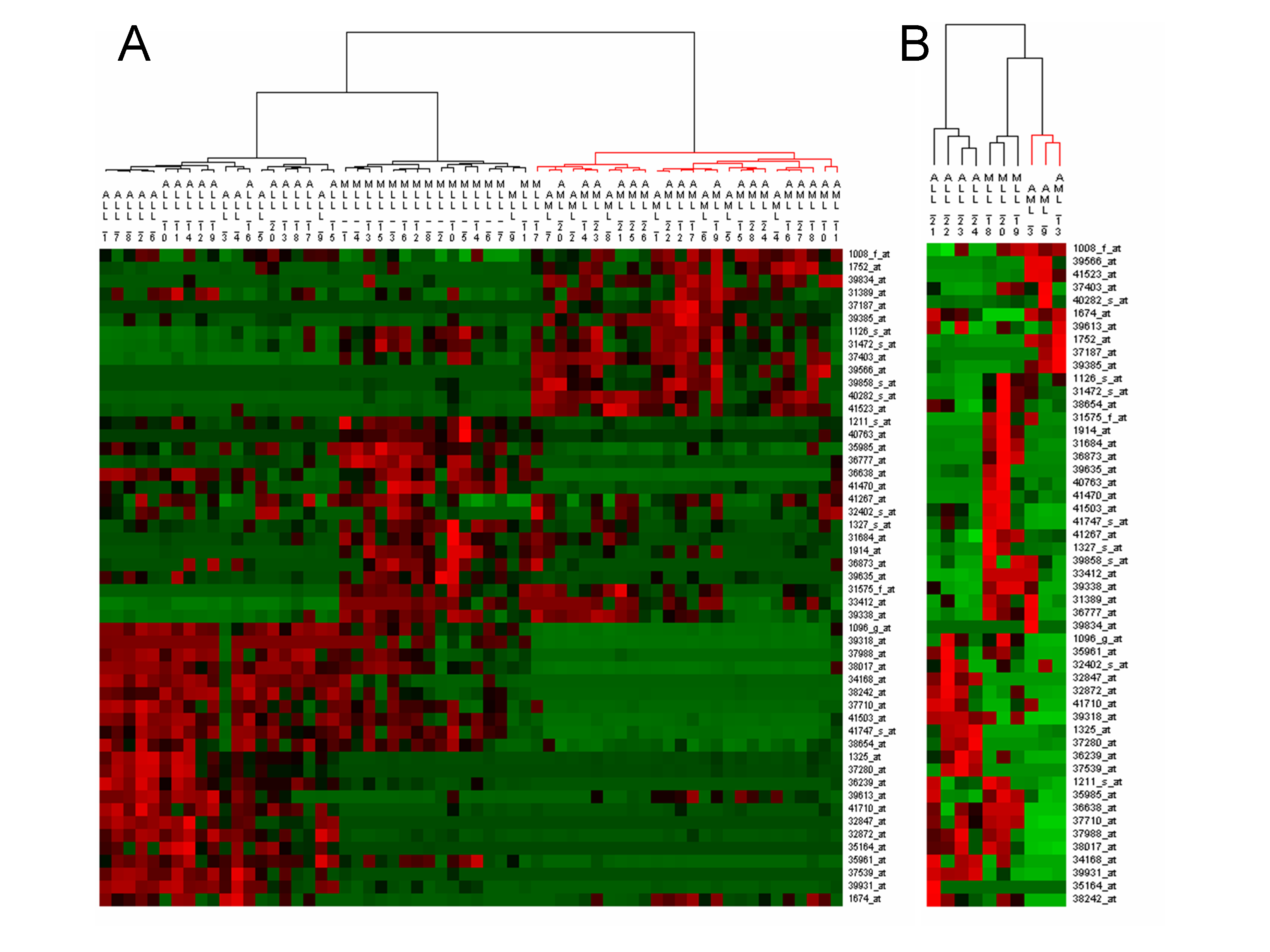

Supplement: Figure S3 — Heatmaps from cluster analysis of reduced training set (A) and test set (B) of top 51 genes chose from dataset of Leukemia_2 by mOPLS-DA models. In training set, only one observation (MLL 17) was misclassified; all observations in independent test set were predicted correctly. (TIF) [file pone.0084253.s003.tif]

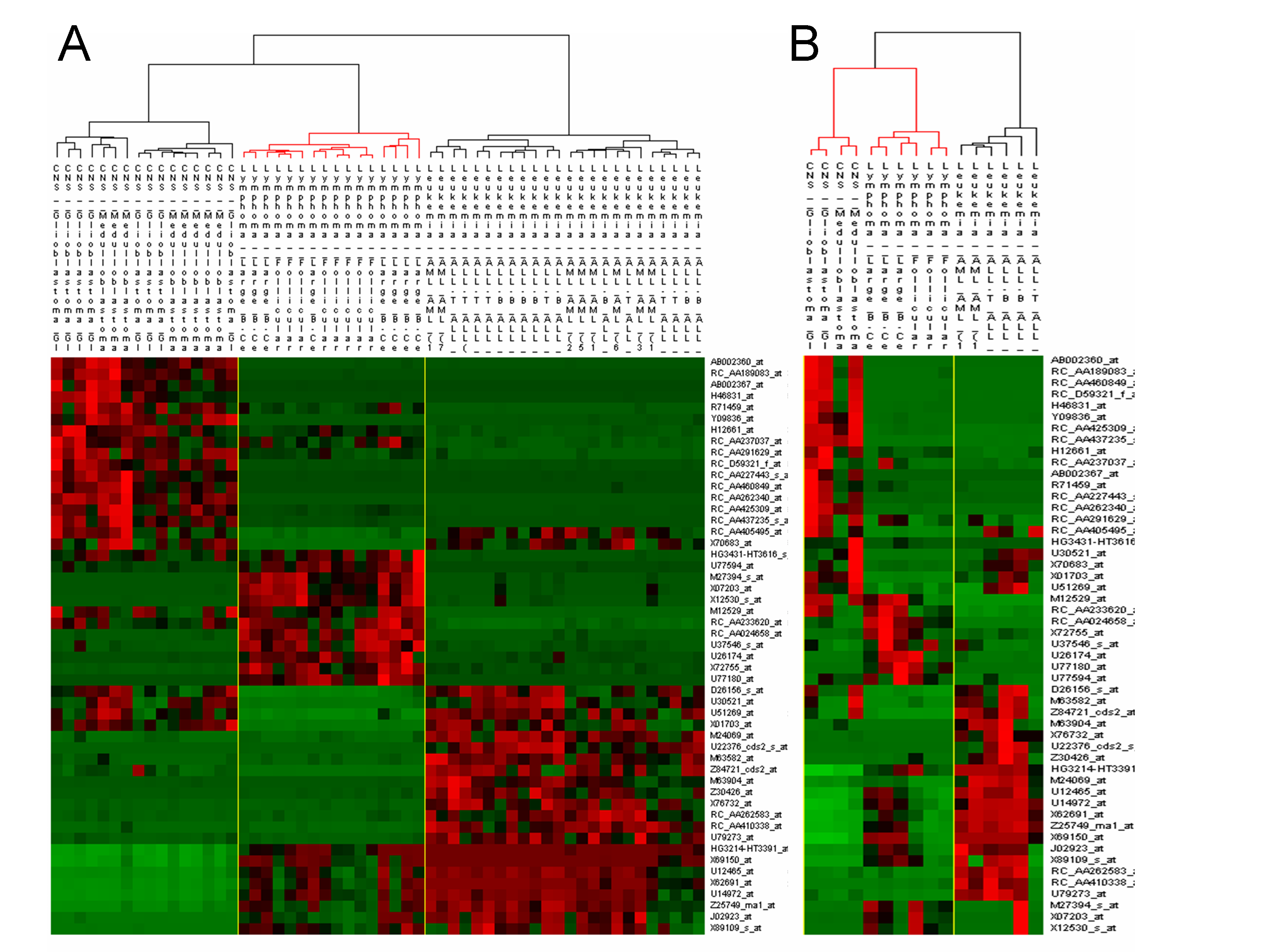

Supplement: Figure S4 — Cluster analysis plot from reduced training set (A) and test set (B) contained top 51 genes selected by mOPLS-DA models using 14_tumour dataset. From these two figures, we can see that all training samples were classified correctly and all test observations were predicted into each class without a mistake. (TIF) [file pone.0084253.s004.tif]
